# Nitrate Storage and Dissimilatory Nitrate Reduction by Eukaryotic Microbes

**DOI:** 10.3389/fmicb.2015.01492

**Published:** 2015-12-22

**Authors:** Anja Kamp, Signe Høgslund, Nils Risgaard-Petersen, Peter Stief

**Affiliations:** ^1^AIAS, Aarhus Institute of Advanced Studies Aarhus UniversityAarhus, Denmark; ^2^Department of Bioscience, Aarhus UniversityAarhus, Denmark; ^3^Department of Biology, Nordic Center for Earth Evolution, University of Southern DenmarkOdense, Denmark

**Keywords:** nitrogen-cycle, nitrate respiration, denitrification, DNRA, diatoms, foraminifers, gromiids, fungi

## Abstract

The microbial nitrogen cycle is one of the most complex and environmentally important element cycles on Earth and has long been thought to be mediated exclusively by prokaryotic microbes. Rather recently, it was discovered that certain eukaryotic microbes are able to store nitrate intracellularly and use it for dissimilatory nitrate reduction in the absence of oxygen. The paradigm shift that this entailed is ecologically significant because the eukaryotes in question comprise global players like diatoms, foraminifers, and fungi. This review article provides an unprecedented overview of nitrate storage and dissimilatory nitrate reduction by diverse marine eukaryotes placed into an eco-physiological context. The advantage of intracellular nitrate storage for anaerobic energy conservation in oxygen-depleted habitats is explained and the life style enabled by this metabolic trait is described. A first compilation of intracellular nitrate inventories in various marine sediments is presented, indicating that intracellular nitrate pools vastly exceed porewater nitrate pools. The relative contribution by foraminifers to total sedimentary denitrification is estimated for different marine settings, suggesting that eukaryotes may rival prokaryotes in terms of dissimilatory nitrate reduction. Finally, this review article sketches some evolutionary perspectives of eukaryotic nitrate metabolism and identifies open questions that need to be addressed in future investigations.

## Introduction

Nitrate is one of the major nutrients for microbial and plant life on planet Earth. It is the most oxidized form of fixed N-compounds, abundant in many aquatic habitats, and of high importance for both assimilatory and dissimilatory nitrogen metabolism.

Nitrate can also be stored inside living cells at concentrations by far exceeding ambient concentrations, a trait that is known for several prokaryotic and eukaryotic phyla (e.g., Dortch et al., 1984; Fossing et al., 1995; McHatton et al., 1996; Schulz et al., 1999; Lomas and Glibert, 2000; Needoba and Harrison, 2004; Risgaard-Petersen et al., 2006; Mußmann et al., 2007; Piña-Ochoa et al., 2010a; Kamp et al., 2011; Bernhard et al., 2012a; Coppens et al., 2014; Stief et al., 2014).

**Figure 1:**
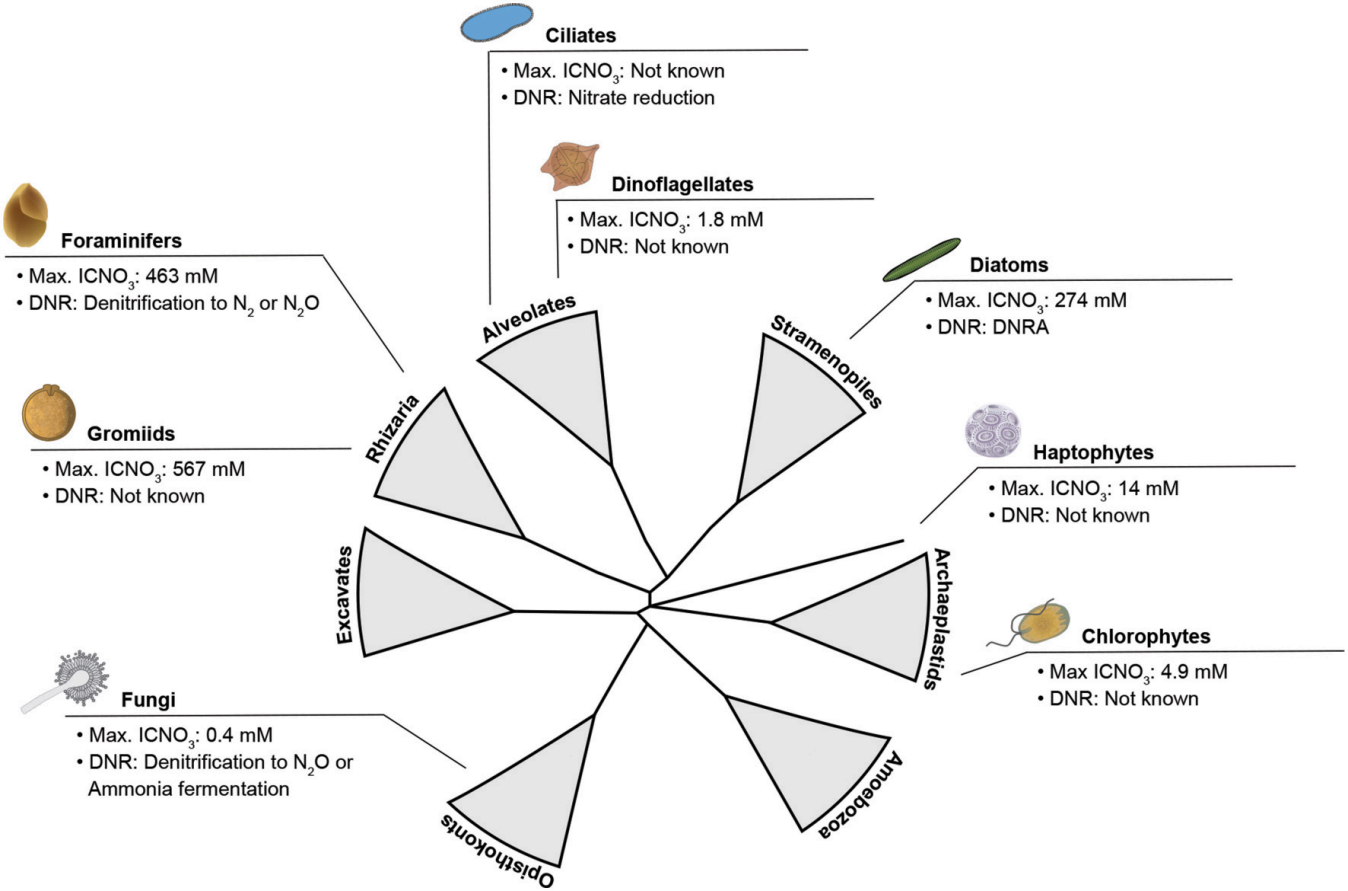
**Schematized eukaryotic tree of life emphasizing the wide distribution of lineages known to store nitrate intracellularly and use it for dissimilation**. Maximum intracellular nitrate concentrations (max. ICNO_3_) and pathways of dissimilatory nitrate reduction (DNR) are given for taxonomic groups tested positive for either trait. DNRA, Dissimilatory Nitrate Reduction to Ammonium. DNR data compiled from Finlay et al. (1983) (ciliates), Shoun and Tanimoto (1991) (fungi), Zhou et al. (2002) (fungi), Risgaard-Petersen et al. (2006) (foraminifers), Kamp et al. (2011) (diatoms). Max. ICNO_3_ data compiled from Dortch et al. (1984) (dinoflagellates, haptophytes), Lomas and Glibert (2000) (chlorophytes), Piña-Ochoa et al. (2010a) (gromiids), Piña-Ochoa et al. (2010b) (foraminifers), Kamp et al. (2011) (diatoms), Stief et al. (2014) (fungi). Tree topology adapted from Worden et al. (2015).

**Figure 2:**
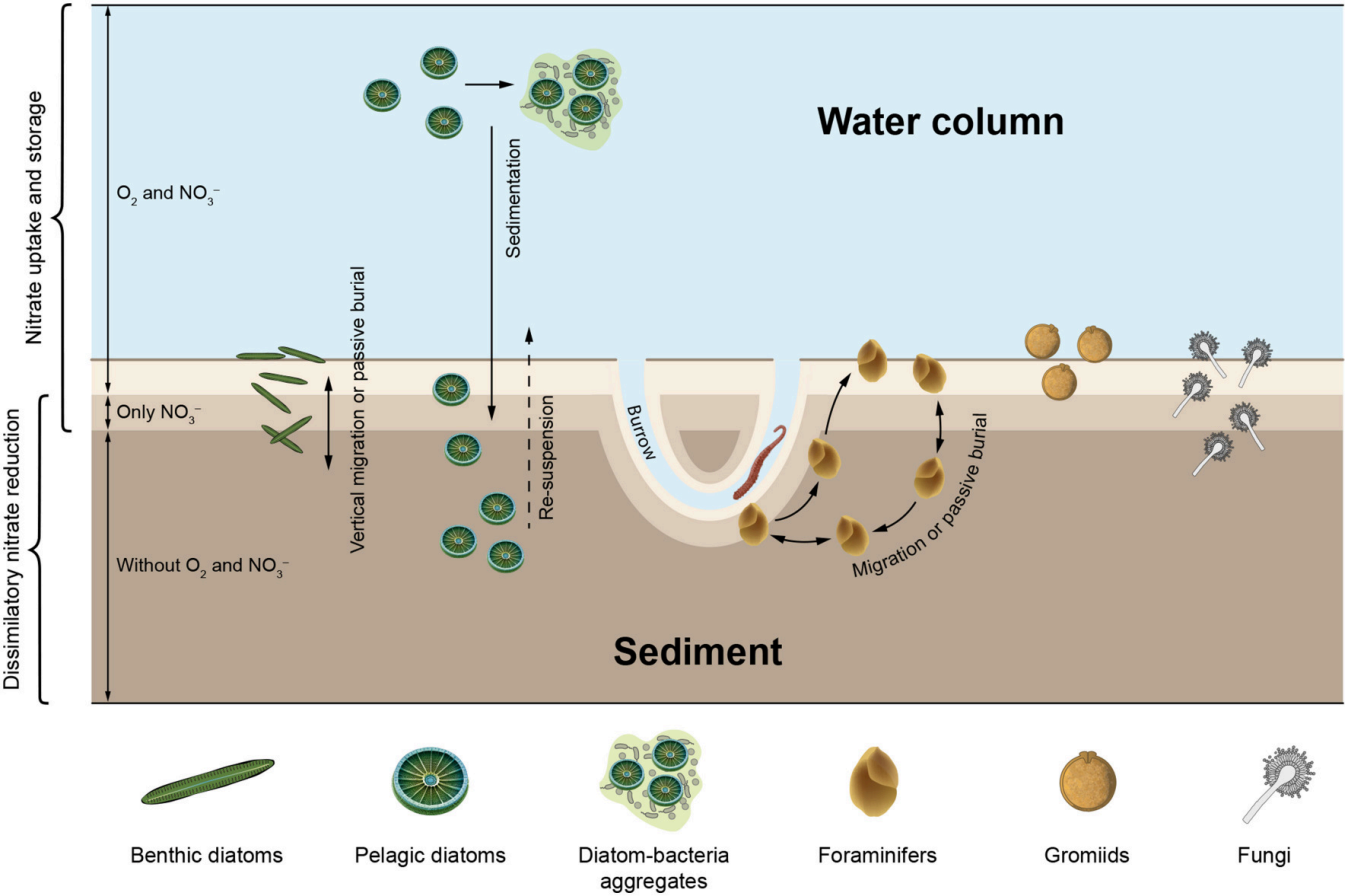
**Marine microbial eukaryotes known to take up ambient nitrate for intracellular storage and/or dissimilatory nitrate reduction drawn in a conceptual scheme of a natural environment with oxic and anoxic compartments**. Organisms and environmental compartments are stylized and not to scale. Scenarios: (1) Benthic diatoms and foraminifers migrate actively up and down between oxic and anoxic sediment layers, or are buried in deep, anoxic sediment layers by e.g., macrofaunal activities, (2) Foraminifers move through different sediment layers and might re-fill their nitrate stores at “hotspots” of nitrate in deeper sediment layers, e.g., macrofaunal burrows, (3) Gromiids reside at the sediment surface or in anoxic subsurface layers, (4) Fungi grow in various sediment layers, (5) Pelagic diatoms sink onto the sediment after phytoplankton blooms and are re-suspended due to spring storms or macrofaunal activities, and (6) Pelagic diatoms are exposed to hypoxic or anoxic conditions inside sinking diatom-bacteria aggregates. Aside from the spatial separation into oxic and anoxic compartments, temporal variation of oxygen availability in the bottom water or inside macrofaunal burrows causes sudden shifts from oxic to anoxic conditions (and back) that may influence nitrate uptake and dissimilatory nitrate reduction by microbial eukaryotes (not shown).

Apparently, the ability to store nitrate intracellularly is widely distributed within the eukaryotic tree of life (Figure 1). Extra- and intracellular nitrate serves for both assimilation and dissimilation. In assimilatory nitrate reduction, ammonium is produced and subsequently incorporated into biomass to build up e.g., proteins and nucleic acids. Dissimilatory nitrate reduction is a process for energy conservation, in which nitrate is used as an electron acceptor in the (near) absence of oxygen (e.g., Fewson and Nicholas, 1961; Strohm et al., 2007; Kraft et al., 2011; Thamdrup, 2012). Dissimilatory nitrate reduction and nitrate storage in particular are physiological life traits that provide microbes with environmental flexibility (i.e., metabolic activity under both oxic and anoxic conditions) and resource independence (i.e., anaerobic metabolism without immediate nitrate supply), respectively. Such life traits are especially important in environments that are temporarily anoxic and/or nitrate-free and they may have developed as a “life strategy” in both prokaryotes and eukaryotes (Figure 2).

Dissimilatory nitrate reduction pathways such as nitrate reduction to nitrite, denitrification, or Dissimilatory Nitrate Reduction to Ammonium (DNRA; Box 1), are well-studied in prokaryotes (i.e., Bacteria and Archaea). Prokaryotes are an integral part of the microbial nitrogen cycle; and due not least to the increasing use of fertilizers and the subsequent pollution of rivers, estuaries, and coastal waters, they have been the focus of many research activities for nearly a 100 years (e.g., Kluyver and Donker, 1926; Fewson and Nicholas, 1961; Zumft, 1997). In contrast, research on eukaryotes that can switch from oxygen to (intracellular) nitrate for anaerobic energy metabolism when (temporarily) exposed to anoxia or hypoxia is still in its infancy. However, the list of eukaryotes so far found to reduce nitrate dissimilatorily includes major global players such as benthic and pelagic marine diatoms, foraminifers, and fungi (Figure 1), which suggests a quantitative impact on nitrogen cycling at least in the marine realm. The first eukaryote that has been found to respire with nitrate in the absence of oxygen was, however, the freshwater ciliate *Loxodes* sp., which survives anoxic conditions in lakes through nitrate reduction to nitrite (Finlay et al., 1983). Later on, soil fungi were shown to be capable of incomplete denitrification to nitrous oxide and nitrate reduction to ammonium (Box 1; Shoun and Tanimoto, 1991; Takaya et al., 1999). Further, the fungus *Aspergillus terreus* isolated from a seasonal oxygen minimum zone (OMZ) in the Arabian Sea was shown to perform DNRA under anoxic conditions (Stief et al., 2014). In 2006, it was demonstrated that certain benthic foraminiferal species perform intracellular accumulation of nitrate, which is subsequently denitrified (Risgaard-Petersen et al., 2006). Only recently, it was discovered that the most important phototrophic group of microbial eukaryotes, the diatoms, also possess a dissimilatory nitrate metabolism. Both a benthic and a pelagic diatom have been shown to perform DNRA after sudden shifts to darkness and anoxia (Kamp et al., 2011, 2013).

Box 1 **Pathways of dissimilatory nitrate reduction in eukaryotes.**

**Denitrification:** Reduction of nitrate via nitrite, nitric oxide, and nitrous oxide to dinitrogen (${\text{NO}}_{3}^{-}\to$ ${\text{NO}}_{2}^{-}\to$ NO → N_2_O → N_2_) with organic or inorganic electron donors. Complete denitrification serves as an efficient N-removal pathway in the environment. Incomplete denitrification may begin and end at various points in the reaction sequence and, for example, produce the greenhouse gas N_2_O.

**Dissimilatory Nitrate Reduction to Ammonium (DNRA):** Reduction of nitrate via nitrite to ammonium (${\text{NO}}_{3}^{-}\to$ ${\text{NO}}_{2}^{-}\to$ ${\text{NH}}_{4}^{+}$) with organic or inorganic electron donors. DNRA does not contribute to N-removal, but rather recycles fixed N in the environment.

**Ammonia Fermentation:** Reduction of nitrate to ammonium coupled to the oxidation of organic electron donors to acetate and substrate-level phosphorylation. Ammonia fermentation has the same effect on N-cycling in the environment as DNRA.

This review summarizes the current knowledge of (i) the ecophysiology of marine microbial eukaryotes that store nitrate intracellularly and thus are potentially involved in dissimilatory nitrate reduction and (ii) the environmental impact of nitrate storage and dissimilatory nitrate reduction by marine microbial eukaryotes on the nitrogen cycle of our oceans. The review further evaluates some evolutionary perspectives, and closes with a more general discussion of open questions that might inspire further research.

## Ecophysiology

### Nitrate storage

#### Diatoms

Among the marine eukaryotic microbes discussed here, intracellular nitrate storage was first described for diatoms, in which intracellular concentrations can by far exceed ambient concentrations (i.e., nitrate in the surrounding seawater). The range of intracellular nitrate concentrations varies according to species and environmental conditions. Many benthic and pelagic diatoms accumulate nitrate intracellularly in concentrations up to a few 100 mM, but concentrations can be as low as zero to a few mM (Dortch et al., 1984; Lomas and Glibert, 2000; Needoba and Harrison, 2004; Høgslund, 2008; Kamp et al., 2011, 2013; Coppens et al., 2014).

Nitrate uptake by diatoms has been shown to be temperature-dependent (decreasing with increasing temperature), is so far documented to occur in oxic conditions only, and uptake rates vary between species (e.g., Raimbault and Mingazzini, 1987; Lomas and Glibert, 1999, 2000; Villareal et al., 1999; Tantanasarit et al., 2013). Lomas and Glibert (2000) measured nitrate uptake rates of 18–310 fmol ${\text{NO}}_{3}^{-}$ cell^−1^ h^−1^ for six different species grown at moderate ambient nitrate concentrations (< 40 μM). This rate can be even higher at very high ambient nitrate concentrations (Tantanasarit et al., 2013).

Intracellular nitrate is generally thought to be located in vacuoles. In the plant vacuole of *Arabidopsis thaliana*, for example, nitrate is accumulated via an ${\text{NO}}_{3}^{-}$/H^+^ exchanger (Martinoia et al., 1981; De Angeli et al., 2006). Evidence for a similar mechanism in unicellular eukaryotes is missing so far.

Nitrate storage in diatoms has long been assumed to serve assimilation exclusively (e.g., Dortch et al., 1984; Lomas and Glibert, 2000), probably because diatoms are mostly found in oxic habitats where nitrate is not needed as an alternative electron acceptor for dissimilation. The first hint for dissimilatory nitrate reduction in diatoms was a correlation between the nitrate storage capacity and the survival time of benthic and pelagic diatoms after sudden shifts to dark and anoxic conditions (Kamp et al., 2011). However, intracellular nitrate is used up within hours and is not replenished from ambient nitrate under anoxic conditions (see below). Lomas and Glibert (1999) also hypothesized that some diatom populations take up nitrate in excess of nutrient requirements because its reduction may serve as a sink for electrons during transient periods of imbalance between light energy harvesting and utilization.

#### Foraminifers

Foraminifers may store nitrate at concentrations >15,000 times the environmental nitrate concentrations (Risgaard-Petersen et al., 2006), probably in vacuoles (Bernhard et al., 2012a), and the measured intracellular nitrate pool varies among nitrate-storing foraminifers from ca. 0.1 mM to >375 mM (Piña-Ochoa et al., 2010a). This variation seems to reflect different physiological and environmental conditions rather than phylogenetic constraints because considerable intraspecific variation is observed among the species that are considered to be nitrate collectors (Piña-Ochoa et al., 2010a; Koho et al., 2011; Bernhard et al., 2012b). The ability to store nitrate at concentrations above environmental concentrations is so far found within the orders Allogromiida, Miliolida, Rotaliida, and Textulariida (Piña-Ochoa et al., 2010a; Bernhard et al., 2012a), and seems to be a common trait for foraminifers from very diverse benthic marine environments, such as OMZs, hypoxic basins, continental slopes, shelf sediments, and coastal sediments (Piña-Ochoa et al., 2010a; Bernhard et al., 2012a). Interestingly, the trait is not restricted to species which often occur in anoxic microhabitats, such as e.g., *Globobulimina turgida*, but is also found in species living in oxic habitats (e.g., *Cassidulina carinata* and *Pyrgo elongata*), and in opportunistic species (e.g., *Bolivina subaenariensis* and *Uvigerina mediterranea*; Piña-Ochoa et al., 2010a). ^15^N labeling experiments performed on Ammonia beccarii, Bolivina argentea, Buliminella tenuata, G. turgida^*^, *Fursenkoina cornuta* and *Nonionella stella* have shown that nitrate is taken up directly from the environment (Risgaard-Petersen et al., 2006; Koho et al., 2011; Bernhard et al., 2012b; Nomaki et al., 2014), and is not produced internally. The mechanism for nitrate uptake as well as the storage mode is at present unknown, but it must involve an active transport system, as nitrate is moved across the cell membrane against a large concentration gradient. From a thermodynamic point of view, the process of nitrate uptake is exergonic and therefore requires an investment of energy by the organism. G. turgida^*^, for instance, may accumulate nitrate internally to well above 10 mM in environments where the environmental concentration is less than 20 μM (Risgaard-Petersen et al., 2006). The Gibbs free energy (ΔG) for nitrate transport across the cell membrane at these conditions is >+15 kJ mol^−1^ ${\text{NO}}_{3}^{-}$ according to equations in Harold (1986). It is evident therefore that nitrate accumulation among foraminifers can only be a sustainable strategy, if required to sustain processes that are essential for the survival of the organism. It has been shown that the nitrate-respiring foraminifer *G. turgida* can survive for up to 56 days of anoxia from respiration of its internal nitrate pool (Piña-Ochoa et al., 2010b), and the building and maintenance of an intracellular nitrate pool might be seen as an insurance that enables the organisms to sustain an active metabolism even when suitable external electron acceptors are absent in the environment (see below).

#### Gromiids

Like the foraminifers, gromiids belong to the Rhizaria (Burki et al., 2010; Sierra et al., 2013), and their intracellular nitrate concentrations can also reach >100 mM, which exceeds the ambient nitrate concentration by several orders of magnitude (Piña-Ochoa et al., 2010a). The ability to accumulate nitrate at these high concentrations appears to be ubiquitous for the gromiids, as it has been found for individuals sampled from hard-bottom substrates, shelf sediments in temperate and arctic regions, as well as in the OMZ along the coastline of Peru (Piña-Ochoa et al., 2010a). The physiology of the gromiids has only been superficially studied and neither the mechanism behind nitrate accumulation, nor its link to any metabolic pathway has been investigated so far. It is possible that the gromiid-nitrate association represents a system that is functionally different from that of benthic foraminifers because gromiids generally are described as surface dwellers, and thus not buried in anoxic sediment layers like many foraminifers (Jepps, 1926; Hedley and Bertaud, 1962; Arnold, 1972; Matz et al., 2008; da Silva and Gooday, 2009; Rothe et al., 2011).

#### Fungi

To date, only a single strain of *A. terreus* isolated from a marine sediment has been shown to store nitrate intracellularly and use it for dissimilatory nitrate reduction mainly to ammonium (Stief et al., 2014). The intracellular nitrate concentration in this strain reached up to 0.4 mM. Unfortunately, intracellular nitrate storage has not been studied in the large number of soil fungi and yeasts capable of dissimilatory nitrate reduction (Takaya et al., 1999; Maeda et al., 2015). Fungi in general, however, do possess cellular vacuoles and nitrate transporters (Klionsky et al., 1990; Navarro et al., 2006) and are able to take up nitrate from the environment at high rates and store it in vacuoles (e.g., 9 nmol ${\text{NO}}_{3}^{-}$ mg^−1^ dry weight min^−1^ in *Aspergillus nidulans*; Unkles et al., 2004).

#### Ciliates

For the only ciliate known to perform dissimilatory nitrate reduction, *Loxodes* sp. (Finlay et al., 1983), intracellular nitrate storage has not been reported.

#### Chlorophytes, dinoflagellates, and haptophytes

Marine phytoplankton belonging to these eukaryotic lineages has mainly been investigated with respect to uptake and assimilation of nitrate and ammonium, but intracellular nitrate storage is also reported occasionally (e.g., Dortch et al., 1984; Lomas and Glibert, 2000). The chlorophyte *Dunaliella tertiolecta* stored nitrate at 2.7–4.9 mM in one study (Lomas and Glibert, 2000), but had intracellular nitrate concentrations below the detection limit in another study (Dortch et al., 1984). Similarly, the dinoflagellate *Amphidinium carterae* stored 0–1.8 mM nitrate (Dortch et al., 1984), while the dinoflagellate *Prorocentrum minimum* did not store nitrate (Lomas and Glibert, 2000). Among the haptophytes, *Isochrysis galbana* stored 0.3–13.9 mM nitrate (Dortch et al., 1984) and *Pavlova lutheri* only stored 0.1–0.2 mM nitrate (Lomas and Glibert, 2000). Clearly, more investigations focusing on intracellular nitrate storage in these lineages are needed.

### Dissimilatory nitrate reduction

#### Diatoms

So far, the benthic diatom *Amphora coffeaeformis* and the pelagic diatom *Thalassiosira weissflogii* have been shown to reduce nitrate dissimilatorily. Both diatom strains perform the pathway Dissimilatory Nitrate Reduction to Ammonium (DNRA), as demonstrated with ^15^N labeling experiments in axenic strains (Kamp et al., 2011, 2013). The DNRA rates of these two diatoms are in the range of 2–3 fmol N cell^−1^ h^−1^ during the first hours after exposure to dark and anoxic conditions. However, DNRA rates become significantly lower after only a few hours, which mirrors the rapid consumption of intracellular nitrate after shifts to darkness and anoxia. Thus, diatoms probably use the intracellular nitrate, and its dissimilatory reduction via DNRA, either for short-term survival or for entering a resting stage.

To date, genes involved in dissimilatory nitrate reduction have not been identified in diatoms, but only in denitrifying soil fungi (see below). Intriguingly, fungi use enzymes that are usually involved in assimilatory nitrate reduction in a dissimilatory mode (Takasaki et al., 2004). This could also be true for diatoms. Assimilatory nitrate reductases, nitrate transporters, and components of a nitrate-sensing system have only recently been identified in diatom genomes (Armbrust et al., 2004; Bowler et al., 2008). Identification of functional genes involved in dissimilatory nitrate reduction in diatoms would provide genetic evidence for this metabolic pathway in diatoms.

#### Foraminifers

Direct measurements of nitrate reduction activity associated with nitrate-storing foraminifers have demonstrated a capacity for complete denitrification of ${\text{NO}}_{3}^{-}$ to N_2_ (Risgaard-Petersen et al., 2006; Høgslund, 2008; Piña-Ochoa et al., 2010a; Bernhard et al., 2012b). Some species (e.g., *Bolivina plicata, Bolivina seminuda, Valvulineria* cf. *laevigata, Stainforthia* sp.), however, seem to lack nitrous oxide reductase and reduce nitrate only to nitrous oxide (Piña-Ochoa et al., 2010a).

At present, denitrification rates for only 11 different species within the Rotaliida order have been determined. The observation of elevated δ^15^N_NO3_ and δ^18^O_NO3_ values in the intracellular nitrate pool within allogromiid foraminifers from the Santa Barbara Basin (Bernhard et al., 2012a) has demonstrated nitrate reduction capacity associated with members of the Allogromiida order, yet rate measurements in this order are still missing. Rates estimated for the Rotaliida with N_2_O-microsensors (Risgaard-Petersen et al., 2006; Høgslund et al., 2008; Piña-Ochoa et al., 2010a,b) or ^15^${\text{NO}}_{3}^{-}$ amendments (Risgaard-Petersen et al., 2006; Bernhard et al., 2012b) fall in the range of 1.7–83 pmol N cell^−1^ h^−1^, and great intraspecific variation is observed. There is a tendency for a log-log relationship between the denitrification rate and biovolume of the organisms, so that large organisms have higher rates than smaller ones, as seen also for foraminiferan cell-specific oxygen respiration rates (Geslin et al., 2011), but the current database is too limited for strong conclusions to be drawn. In general, individual denitrification rates are much lower than the corresponding oxygen respiration rates (Piña-Ochoa et al., 2010a) and it has been suggested that denitrification is an auxiliary metabolism used for cell maintenance, food collection, and locomotion during temporary stays in oxygen-free environments, whereas oxygen might be required for growth and reproduction (Piña-Ochoa et al., 2010a,b).

The genes behind the foraminiferan denitrification pathway have not been elucidated. It has, however, been shown with microscopy (Risgaard-Petersen et al., 2006) and experiments applying bacteria-specific antibiotics to denitrifying foraminifers (Bernhard et al., 2012b) that for some species (e.g., *B. argentea* and G. turgida^*^) the foraminifers themselves, and not only the associated prokaryotes, are performing the denitrification reaction. Denitrification in a nitrate-storing allogromiid foraminifer from the Santa Barbara Basin, however, appears to be performed by prokaryotic endobionts and not the eukaryote, as demonstrated by sequence analyses and GeneFISH (Bernhard et al., 2012a). Given the widespread distribution of nitrate-accumulating and denitrifying foraminifers within diverse phylogenetic orders, specific investigations of each group are needed, at best on the genomic level, to confirm or reject the presence of eukaryotic denitrification. It is obvious from the Santa Barbara study that a capacity for nitrate accumulation is not necessarily coupled to a capacity of the eukaryote to utilize this directly for energy conservation through e.g., denitrification.

#### Fungi

The best-studied fungi species capable of dissimilatory nitrate reduction are the two soil-living plant pathogens *Fusarium oxysporum* and *Cylindrocarpon tonkinense* (Shoun and Tanimoto, 1991; Usuda et al., 1995). The majority of terrestrial fungi, some ectomycorrhizal fungi, and many of the yeast strains screened since the initial discovery of “fungal denitrification” also tested positive for this trait (Tsuruta et al., 1998; Prendergast-Miller et al., 2011; Mothapo et al., 2013; Maeda et al., 2015). A key feature of “fungal denitrification” is the absence of the last reduction step of the denitrification pathway, which makes fungi very important nitrous oxide (N_2_O) producers in soils (Laughlin and Stevens, 2002; Crenshaw et al., 2008; Chen et al., 2014). Fungi isolated from aquatic ecosystems have received much less attention in terms of dissimilatory nitrate reduction, and conclusive experiments with ^15^${\text{NO}}_{3}^{-}$ labeling have been made for only one single strain of *A. terreus* isolated from sediment in the seasonal oxygen minimum zone of the Arabian Sea (Stief et al., 2014). This strain also has a high N_2_O yield (approximately 15% of the total amount of N produced), but the main product of its nitrate reduction activity is ammonium (up to 83%, equivalent to 175 nmol N g^−1^ protein h^−1^), which is also the case for a number of soil fungi (Zhou et al., 2002). The underlying metabolic pathway has been termed “ammonia fermentation” a process which couples the oxidation of ethanol to acetate, and the reduction of nitrate to ammonium, to substrate-level phosphorylation (Box 1; Takaya, 2009). Thus, two pathways of dissimilatory nitrate reduction have evolved in fungi, apparently also within individual species (e.g., *F. oxysporum*; Zhou et al., 2002). The prevalence of either pathway is controlled by ambient oxygen levels, with hypoxic and anoxic levels triggering “fungal denitrification” and “ammonia fermentation,” respectively (Takaya, 2009).

Key genes of “fungal denitrification” have been identified and sequenced (Kizawa et al., 1991; Kim et al., 2009). Nitrite reduction to nitric oxide (NO) is mediated by a copper-containing nitrite reductase (*NirK*), while the reduction step from NO to N_2_O is mediated by the cytochrome *P450* nitric oxide reductase (*P450nor*). Nitrous oxide reductases are generally absent in fungi, which explains why N_2_O instead of N_2_ is the final product of “fungal denitrification” (Takaya, 2009). Dissimilatory nitrate reductases can be present in some denitrifying fungi species, but are less well-characterized than *NirK* and *p450nor* (Takaya, 2009). Hence, the minimal denitrification pathway in fungi only comprises the two-step reduction of nitrite to N_2_O. The stepwise reduction of nitrate to ammonium in fungal “ammonia fermentation” is apparently mediated by assimilatory nitrate (*NiaD*) and nitrite reductases (*NiiA*) used in a dissimilatory context, i.e., it is coupled to the fermentation of ethanol to acetate (Takasaki et al., 2004). Meanwhile, several primer sets for the fungal *NirK* and *p450nor* have been developed and used for screening fungal isolates for their capability of dissimilatory nitrate reduction (Kim et al., 2010; Maeda et al., 2015; Mothapo et al., 2015; Wei et al., 2015). The availability of these primer sets will enable the detection of denitrifying fungi in environmental samples without prior isolation, cultivation, and functional testing.

#### Ciliates

The freshwater ciliate *Loxodes* sp. survives anoxic conditions in lakes through dissimilatory nitrate reduction to nitrite (Finlay et al., 1983; Aleya et al., 1992). A link was made between the anaerobic metabolism of the ciliate and a higher number of mitochondria per cell and a greater surface area of cristae inside the mitochondria compared to specimens exposed to oxic conditions (Finlay et al., 1983; Finlay, 1985). To date, the gene encoding the nitrate reductase has not been identified.

#### Chlorophytes, dinoflagellates, and haptophytes

None of the aforementioned nitrate-storing representatives of these eukaryotic lineages (see Nitrate Storage) has been tested for dissimilatory nitrate reduction under anoxic conditions so far.

### Habitats and life style

Dissimilatory nitrate reduction by prokaryotes and eukaryotes typically occurs in environments in which the availability of oxygen and nitrate is variable in space and time. Stable environments are spatially structured into zones with/without oxygen and/or nitrate availability, while dynamic environments are temporally structured into phases with/without oxygen and/or nitrate availability. In aquatic ecosystems, such conditions can be found in sediments, around animal burrows in sediments, in the root zone of aquatic plants, in low-oxygen water bodies, and inside sinking organic aggregates.

In sediments with stable redox stratification, oxygen, as the most favorable electron acceptor in terms of energy, is consumed within the top few millimeters (Revsbech et al., 1980). Nitrate penetrates slightly deeper into the sediment where it is used as an alternative electron acceptor when oxygen is depleted (Sweerts and de Beer, 1989). Microbes that are able to store nitrate intracellularly may thrive well below the nitrate penetration depth, but need to fill up their nitrate stores occasionally. Large sulfur bacteria couple vertical migration behavior to uptake and storage of nitrate at the sediment surface and dissimilatory use of intracellular nitrate deeper in the sediment (e.g., Fossing et al., 1995). Benthic foraminifers and diatoms, both of which are capable of migrating inside sediments, can be abundant well below the nitrate penetration depth (Figure 2; Risgaard-Petersen et al., 2006; Stief et al., 2013). Benthic diatoms exhibit a vertical migration rhythm that is coupled to diurnal and tidal cycles (Consalvey et al., 2004), while foraminifers migrate more erratically or directed to oxygen gradients (Alve and Bernhard, 1995; Geslin et al., 2004; Koho et al., 2011). As a consequence of their migration behavior, benthic diatoms and foraminifers are exposed to elevated ambient nitrate and oxygen levels whenever they reach the sediment surface. Deeper in the sediment where they find shelter from predation and erosion (Kingston, 1999), diatoms and foraminifers face the absence of ambient nitrate and oxygen.

Oxygen and nitrate concentration gradients in sediments can experience rapid and pronounced changes caused by disturbance events. Short-term oxygen and nitrate pulses occur in animal burrows that reach deep into anoxic sediment layers and are intermittently irrigated with oxygen- and nitrate-rich surface water (Kristensen et al., 1991; Wenzhöfer and Glud, 2004). During the resting phase of the animals, oxygen is depleted faster than nitrate, which allows for short-term dissimilatory nitrate reduction in the immediate surrounding of the burrow (Stief and de Beer, 2006). The root zone of aquatic plants exhibits oxygen and nitrate dynamics that are similar to animal burrows, albeit due to periodic changes in the photosynthetic activity of the plant (Frederiksen and Glud, 2006). In the light, roots release oxygen into the surrounding sediment and stimulate nitrate production by microbial nitrification, while in the dark, nitrate is depleted due to dissimilatory nitrate reduction activities (Risgaard-Petersen and Jensen, 1997). It has been suggested that the nitrate-storing and sulfide-oxidizing *Thioploca ingrica* are able to exploit nitrate pulses in animal burrows to fill up their nitrate stores (Høgslund et al., 2010). Benthic diatoms and foraminifers are often abundant in this dynamic microenvironment in which conditions conducive to nitrate uptake and dissimilatory nitrate reduction alternate (Alve and Bernhard, 1995; Steward et al., 1996).

Hypoxic or anoxic water bodies in which nitrate is available may also host dissimilatory nitrate reduction mediated by eukaryotes. The ciliate *Loxodes* sp. is abundant just below the oxic-anoxic interface of stratified lakes, where it reduces nitrate dissimilatorily to nitrite (Finlay et al., 1983; Aleya et al., 1992). Marine pelagic diatoms can move up and down through the water column by controlling their buoyancy (Armbrust, 2009) and are thereby exposed to varying ambient nitrate and oxygen levels (Villareal et al., 1993). Rapid and large-scale transport of diatoms through the water column of the oceans occurs when diatoms and bacteria aggregate to form “marine snow” (Thornton, 2002). Sinking organic aggregates also exhibit internal gradients of oxygen concentration due to microbial respiration and transport limitation of oxygen (Ploug et al., 1997). Under dark conditions (i.e., at night or when aggregates sink out of the photic zone) the center of aggregates may become anoxic, which allows for dissimilatory nitrate reduction (Klawonn et al., 2015). Pelagic diatoms finally sink onto the seafloor, where they still host a large inventory of intracellular nitrate (Lomstein et al., 1990) and may survive in dark, anoxic sediment layers for decades (Härnström et al., 2011), thus far longer than the intracellular nitrate pool would last.

## Environmental impact

### Inventory of intracellular nitrate pools

**Figure 3:**
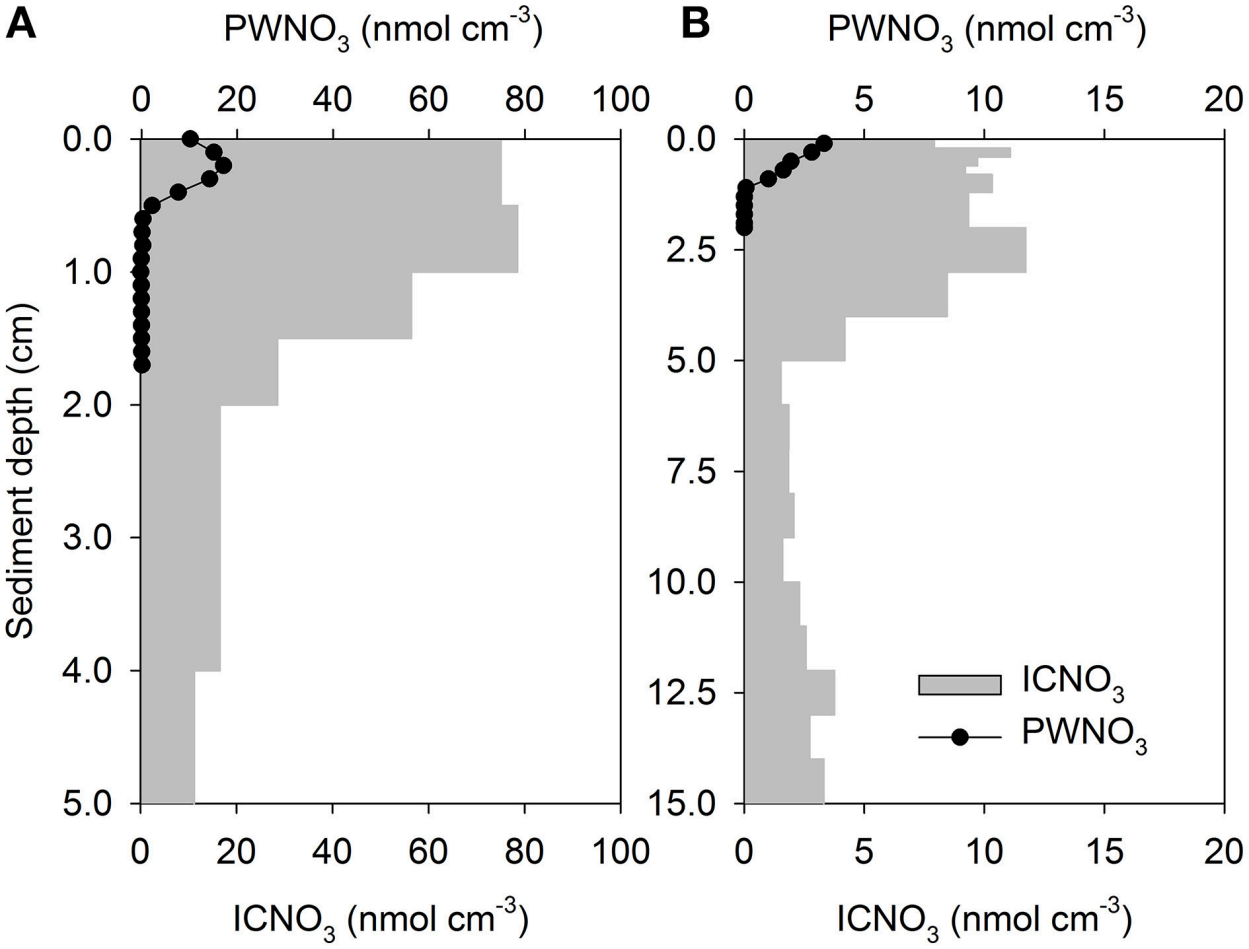
**Vertical profiles of porewater nitrate (PWNO_**3**_) and intracellular nitrate (ICNO_**3**_) in (A) foraminifer-inhabited and (B) diatom-inhabited marine sediments**. Nitrate concentrations are expressed per cm^3^ of sediment. Note different scales. Data compiled from **(A)** Risgaard-Petersen et al. (2006) and **(B)** Heisterkamp et al. (2012).

**Figure 4:**
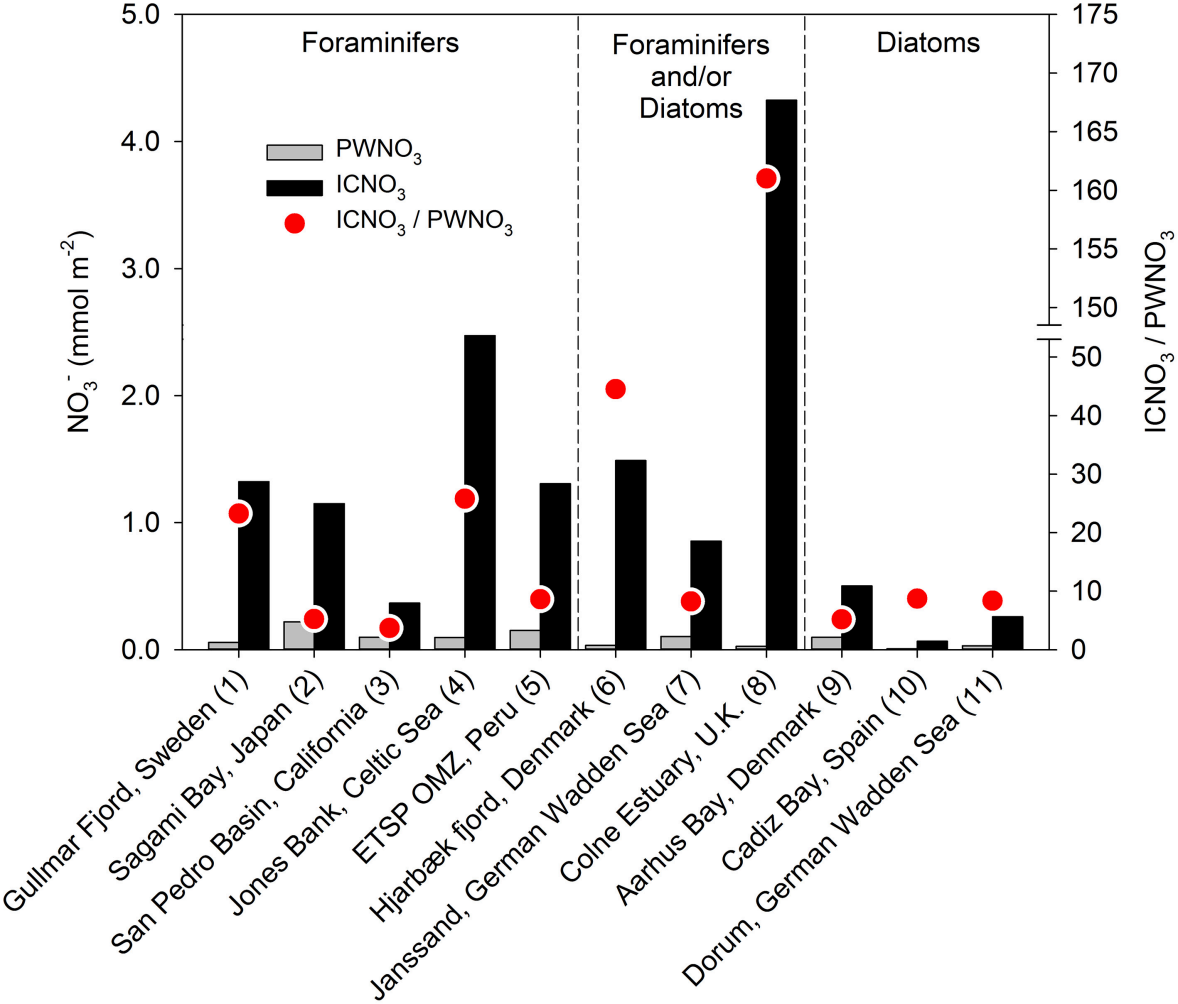
**Inventories of porewater nitrate (PWNO_**3**_) and intracellular nitrate (ICNO_**3**_) in various marine sediments**. Only studies in which the sedimentary ICNO_3_ pool is ascribed to nitrate-storing foraminifers and/or diatoms are considered. Data compiled from (1) Risgaard-Petersen et al. (2006), (2) Glud et al. (2009), (3) Prokopenko et al. (2011), (4) Larsen et al. (2013), (5) Glock et al. (2013), (6) Høgslund et al. (2010), (7) Marchant et al. (2014), (8) Papaspyrou et al. (2014), (9) Lomstein et al. (1990), (10) García-Robledo et al. (2010), and (11) Heisterkamp et al. (2012). Details on data extraction can be found in Table S1.

The presence of nitrate-storing eukaryotes in sediments leads to large inventories of nitrate that vastly exceed the porewater nitrate contents in some environments, equivalent to what can be observed in sediments colonized with the sulfide-oxidizing bacteria *Thioploca* sp. or *Beggiatoa* sp. (Jørgensen and Gallardo, 1999; Sayama, 2001). These intracellular nitrate pools (ICNO_3_ pools) are measured with a diverse set of methods, such as freeze-thaw cycling, boiling, whole-core squeezing, and centrifugation of environmental samples, which all aim at lysing nitrate-storing cells (e.g., Lomstein et al., 1990; Risgaard-Petersen et al., 2006; Prokopenko et al., 2011; Larsen et al., 2013). In sediments from the Gullmar Fjord, Sweden, for instance, nitrate dissolved in the sediment porewater (PWNO_3_) accounted for less than 4% of the total nitrate pool (Risgaard-Petersen et al., 2006). The remaining nitrate, as extracted by boiling the sediment, was most likely present in eukaryotic cells since neither Beggiatoa, nor Thioploca was present. The sediment was inhabited by the nitrate-storing foraminifer *Globobulimina pseudospinescens*, and the cell-bound nitrate was significantly correlated with the abundance of this organism. However, the intracellular nitrate pool of *G. pseudospinescens* only accounted for approximately 20% of total nitrate in the sediment, leaving open the possibility that other nitrate-storing foraminifers or diatoms, gromiids, and fungi were present. Settled phytoplankton with nitrate-storing representatives among the diatoms, chlorophytes, dinoflagellates, and haptophytes may contribute to the sedimentary ICNO_3_ pool that is not accounted for by benthic eukaryotes and prokaryotes. Meanwhile, large sedimentary ICNO_3_ pools were ascribed to the presence of benthic foraminifers in various marine ecosystems (Figures 3A, 4; Table S1). Intracellular nitrate pools were ~4–26 times larger than porewater nitrate pools (Figure 4; Glud et al., 2009; Prokopenko et al., 2011; Glock et al., 2013; Larsen et al., 2013). In three additional studies, it was assumed that both foraminifers and/or diatoms contribute to the ICNO_3_ pool in marine sediments (Figures 3A,B, 4; Table S1; Høgslund et al., 2010; Marchant et al., 2014; Papaspyrou et al., 2014).

In a number of coastal sediments, the total ICNO_3_ pool has been exclusively assigned to microalgae, in particular to pelagic diatoms that have settled onto the sediment surface (Lomstein et al., 1990) and to benthic diatoms that reside in intertidal sediments (García-Robledo et al., 2010; Heisterkamp et al., 2012; Stief et al., 2013). In natural settings, diatom-associated ICNO_3_ is diagnosed as a congruent distribution of ICNO_3_ and fucoxanthin, the marker pigment of diatoms (Stief et al., 2013). In diatom-dominated sediments, the ratio of ICNO_3_-to-PWNO_3_ tends to be lower (~5–9) than in foraminifer-dominated sediments (Figure 4; Table S1), but more data need to be collected to confirm this preliminary observation. The pronounced seasonality in intertidal communities of the temperate zone also entails seasonal changes of the diatom-associated ICNO_3_ pool with high and low values in the cold and warm season, respectively (Stief et al., 2013). It is currently not known whether other nitrate-storing eukaryotes show similar seasonal variation of their ICNO_3_ contents.

The *in situ* turnover of the sedimentary ICNO_3_ pool is indicated by elevated δ^15^N_NO3_ and δ^18^O_NO3_ values (Prokopenko et al., 2011; Bernhard et al., 2012b). These isotope ratios further increase when isolated foraminifers are incubated under anoxic conditions, which confirms dissimilatory nitrate reduction activity fueled by ICNO_3_ (Bernhard et al., 2012b). Estimated turnover times of ICNO_3_ vary between ~12 h and ≥1 month (Risgaard-Petersen et al., 2006; Høgslund, 2008; Glud et al., 2009; Bernhard et al., 2012b), which is considerably slower than the turnover of PWNO_3_ of only 2–4 h, which is determined in sediments inhabited by foraminifers (Glud et al., 2009; Larsen et al., 2013). This slow turnover of ICNO_3_ by foraminifers and other nitrate-storing eukaryotes has implications for rate measurements based on ^15^${\text{NO}}_{3}^{-}$ incubations (Høgslund, 2008). The labeled and non-labeled nitrate pools may not readily mix within short incubation times, which leads to a significant underestimation of benthic denitrification rates determined with the isotope pairing technique (Nielsen, 1992). Additionally, the unintended release of ICNO_3_ from eukaryotic cells into the sediment porewater due to crude extraction techniques simulates concentration peaks that might be mistaken for nitrate production zones.

### Estimates of eukaryotic dissimilatory nitrate reduction

**Figure 5:**
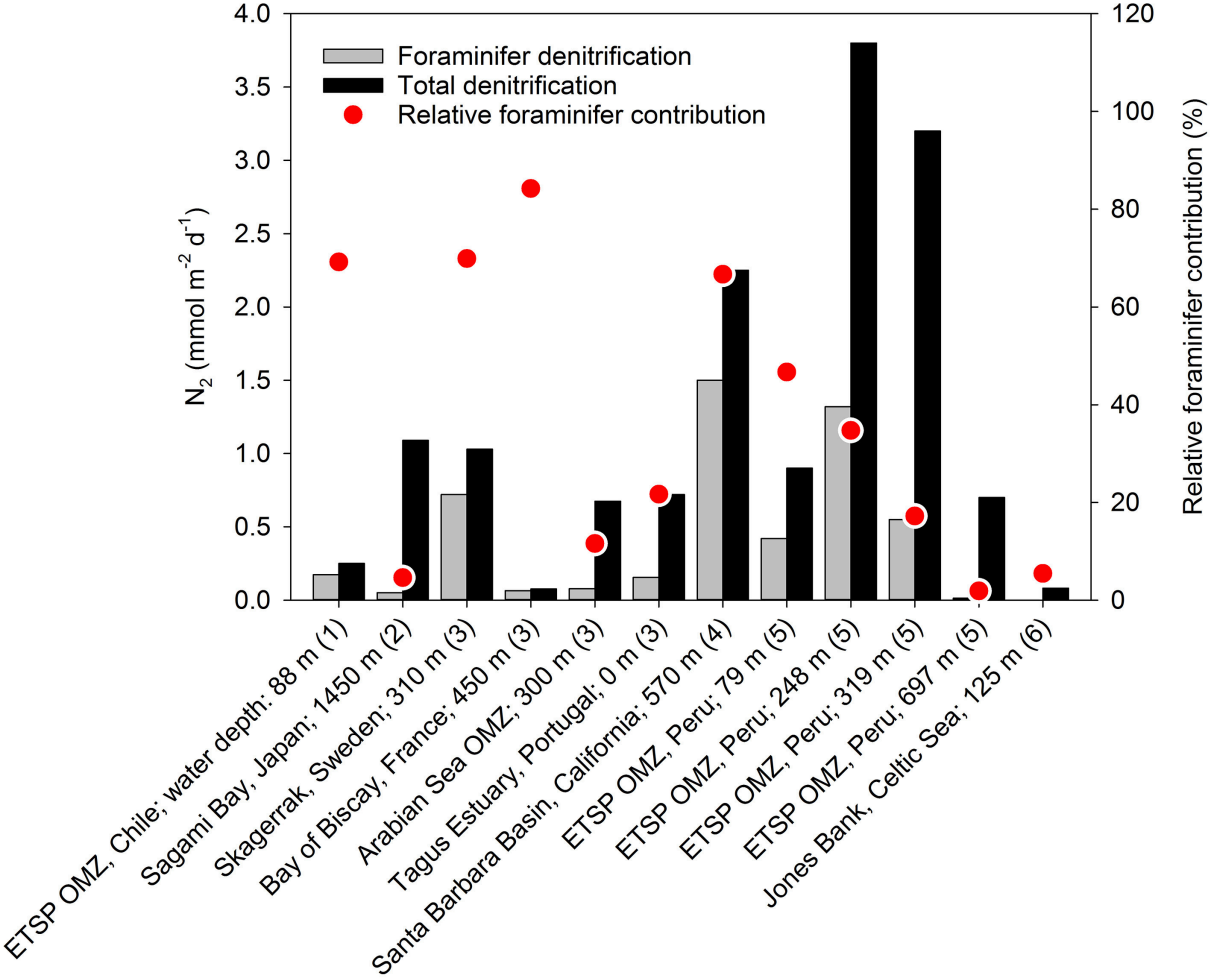
**Foraminiferan denitrification and total denitrification in various benthic marine environments**. Data compiled from (1) Høgslund et al. (2008), (2) Glud et al. (2009), (3) Piña-Ochoa et al. (2010a), (4) Bernhard et al. (2012b), (5) Glock et al. (2013), and (6) Larsen et al. (2013).

The quantitative role of eukaryote-associated nitrate reduction has only been addressed for foraminifers. The contribution of foraminiferal denitrification to the total loss of combined nitrogen from marine sediments has been estimated for various benthic settings (Høgslund, 2008; Glud et al., 2009; Piña-Ochoa et al., 2010a; Bernhard et al., 2012b; Glock et al., 2013). Apparently, foraminifers may contribute substantially to benthic denitrification and in some environments they even surpass the contribution from prokaryotes (Figure 5).

Foraminiferan denitrification is in general estimated from the *in situ* abundance of live foraminifers and laboratory-based estimates of denitrification rates for individual species, whereas total denitrification is estimated from ^15^N-enrichment studies, from porewater profiles of nitrate, or from analyzing the distribution of δ^15^N_NO3_ in natural settings (Groffman et al., 2006). This approach involves a high degree of uncertainty as (i) there is only limited information about the diversity of foraminiferal denitrification activity (data from only 11 species are available), (ii) the cell-specific activity is typically measured at conditions far from natural environmental conditions, and (iii) foraminiferal denitrification is typically not included in standard techniques used for measuring total denitrification (see above). Therefore, present reports on foraminiferal contribution to benthic denitrification activity should be considered as preliminary attempts. There is certainly a need for methodologies that capture the *in situ* denitrification activity mediated by eukaryotes vs. prokaryotes.

## Evolution

The last decade of research has shown that the eukaryotes have their evolutionary roots in a largely anoxic ocean (Anbar and Knoll, 2002; Martin et al., 2003). Pronounced atmospheric oxygenation occurred around 2.4 to 2.1 billion years ago as a consequence of oxygenic photosynthesis, and is named the Great Oxidation Event (GOE). For a few years, it has been debated whether the first production and local accumulation of oxygen might already have happened 2.7–3.2 billion years ago (Brocks et al., 1999; Lyons et al., 2014; Satkoski et al., 2015). Even after the GOE, however, it was not until ~600 million years ago that widespread oxygenation of the deep ocean occurred (Canfield, 1998; Canfield et al., 2008; Lyons et al., 2014). During this transition period of ~1.8 billion years, oxygen was first produced in oceanic microhabitats, which allowed nitrification, the critical aerobic pathway in the nitrogen cycle that produces nitrate, to proceed. Thus, nitrate was present in the oxic microniches of the Proterozoic ocean, and became available for dissimilatory nitrate reducers living on the edge of the nitrate production zones (Fennel et al., 2005; Canfield et al., 2010).

The occurrence of dissimilatory nitrate reduction in very distantly related eukaryotes (Figure 1) raises the question whether the genes involved were present in the single eukaryotic common ancestor that emerged in the largely anoxic Proterozoic ocean, or, alternatively, whether the dissimilatory nitrate reduction pathways we observe today exhibit multiple origins. Neither of the two hypotheses excludes the mitochondrion as the location for eukaryotic nitrate dissimilation. The mitochondrial proteome is a mosaic assemblage of proteins where some are traced to the last mitochondrial ancestor within the Alphaproteobacteria, and some have been acquired from other prokaryotes and eukaryotes through the course of evolution (Gray, 2015).

There is ample evidence that contemporary mitochondria-related organelles are derived from the same ancestral organelle (Mentel and Martin, 2008; van der Giezen, 2011) and the genes involved in dissimilatory nitrate reduction could have been introduced during this event. The suggested ancestor of the mitochondria within the Alphaproteobacteria (Yang et al., 1985; Williams et al., 2007; Gray, 2015) is a representative of a metabolically versatile class that contains facultative anaerobic species capable of running both aerobic respiration and denitrification, e.g., *Paracoccus denitrificans*, which is discussed as a candidate for the protomitochondrion (John and Whatley, 1975; Gray, 2015). The metabolic blueprint provided by the protomitochondrial Alphaproteobacteria has been extensively modified and today we observe diverse functions and biochemical pathways tied to mitochondria in different eukaryotic lineages (Müller et al., 2012). Investigations of mitochondrial proteomes in nitrate-reducing eukaryotes may show diverse routes of acquisition of the proteins involved in the pathways. This seems to be the case at least for the fungi.

The fungal nitrate respiration is tied to the mitochondrion and details of the denitrification pathway of the soil fungus *F. oxysporum* have largely been resolved. The conversion of nitrite to nitric oxide is coupled to the mitochondrial electron transport chain and ATP synthesis and involves a copper-containing nitrite reductase, *NirK* (Kobayashi et al., 1996; Kim et al., 2009; Long et al., 2015). Because the distribution of the eukaryotic *NirK* gene is systematic and follows the eukaryotic phylogeny, it is suggested that the trait of nitrite reduction evolved from a single ancestor and was carried into the eukaryotic domain in the endosymbiotic event leading to the evolution of the mitochondrion (Kim et al., 2009; Shoun et al., 2012). Apparently though, the fungal reduction of nitric oxide to nitrous oxide has a different origin (Shoun et al., 2012). This reduction step is mediated by a *P450* nitric oxide reductase that is found in both the mitochondria and in the cytoplasm, and it seems that this part of the denitrification pathway has been acquired by lateral gene transfer (Kizawa et al., 1991).

Moving from the fungi in the Opisthokonta to the ciliates in the Alveolata, we also find that dissimilatory nitrate reduction by the ciliate *Loxodes* sp. is presumably affiliated with the mitochondria (Finlay et al., 1983; Finlay, 1985). Gene sequences of this nitrate reductase are, however, not available and it is, at the moment, not possible to draw conclusions about the origin of the trait.

Nitrate accumulation is dispersed throughout the foraminiferan phylogeny including allogromiid species, which likely evolved in the Neoproterozoic anoxic ocean and are considered to form a basal order within the foraminifers (Pawlowski et al., 2003). This might suggest that nitrate accumulation was present in the most recent common ancestor of foraminifers, albeit lost in some lineages. Usage of the intracellular nitrate may have evolved differently among foraminiferal lineages, since data at present show that the allogromiids share their nitrate with endobionts (Bernhard et al., 2012a), whereas some more recently evolved rotaliids invoke it in their own dissimilatory metabolic pathways (Risgaard-Petersen et al., 2006).

Genes for dissimilatory nitrate reduction in diatoms are not known, but diatoms have remarkable genomes with traces of multiple plastid endosymbiotic events allowing migration of genes from the plastid endobiont into the genome (Prihoda et al., 2012). A large number of genes also seem to be derived from bacteria by lateral gene transfer (Bowler et al., 2008; Armbrust, 2009), including genes for nitrite reductases that are targeted at the mitochondria (Allen et al., 2006). This knowledge of the chimeric diatom genomes paves the way for speculations on lateral transfers of genes involved in the dissimilatory use of nitrate, but the examination of such hypotheses requires further sequencing and bioinformatics efforts.

The extensive lack of information on the genes and enzymes driving dissimilatory nitrate reduction among the eukaryotes also means that we cannot close in on the time of its evolutionary origin. It can be established, however, that an anaerobic energy metabolism coupled to nitrate reduction was possible in the environmental settings at the time of origin of ascomycete fungi (Lücking et al., 2009; Prieto and Wedin, 2013) and foraminifers (Pawlowski et al., 2003; Groussin et al., 2011). Diatoms evolved only 250 million years ago (Sims et al., 2006; Armbrust, 2009) and existing hypotheses of diatom origins tend to agree that the pre-diatom or “Ur-diatom” developed in shallow marine (and thus more oxygenated) environments (Sims et al., 2006; Medlin, 2011). It will be interesting to follow the evolutionary path of dissimilatory nitrate reduction among eukaryotes when more sequence data becomes available.

## Open questions and further directions

The study of nitrate storage and dissimilatory nitrate reduction by eukaryotic microbes is still in its infancy. Finding nitrate reduction to nitrite by *Loxodes* sp. and denitrification by fungi were early milestones reached a few decades ago. More recently though, this research area has gained momentum by the discovery that diatoms and foraminifers are also capable of dissimilatory nitrate reduction coupled to intracellular nitrate storage. Future research activities should address open questions regarding the (i) phylogenetic diversity, (ii) physiology and genetics, and (iii) *in situ* importance of eukaryotic nitrate storage and dissimilatory nitrate reduction.

The known occurrence of nitrate storage and reduction in distant eukaryotic lineages (Figure 1) suggests that these physiological traits are even more widespread among eukaryotes than previously thought. In particular, those lineages for which nitrate storage has already been documented might be “hot candidates” for performing dissimilatory nitrate reduction under anoxic conditions, e.g., gromiids and dinoflagellates (Dortch et al., 1984; Piña-Ochoa et al., 2010a). There is a growing interest in microbial eukaryotes adapted to life under low-oxygen conditions (e.g., Stoeck et al., 2009; Edgcomb et al., 2011; Müller et al., 2012; Bernhard et al., 2014; Parris et al., 2014). An obvious research strategy is, thus, to test known and novel eukaryotic lineages from low-oxygen environments for their ability to store nitrate intracellularly (Step 1) and use nitrate as an alternative electron acceptor (Step 2).

Some fundamentals of the physiology, biochemistry, and genetics of eukaryotic nitrate storage and reduction are still unknown. While ^15^N labeling experiments have proven intracellular nitrate as an alternative electron acceptor in dissimilatory processes in fungi, foraminifers, and diatoms, there is still a role for intracellular nitrate in assimilation. The exact partitioning of intracellular nitrate between dissimilation and assimilation remains to be investigated in the diverse nitrate-storing and nitrate-reducing eukaryotes. Further unknowns concern the mechanism and energy requirements of nitrate uptake, the intracellular compartment of nitrate storage, and the spectrum of electron donors used for dissimilatory nitrate reduction (e.g., organic vs. inorganic, external vs. storage compounds).

The identification of genes that encode for enzymes involved in dissimilatory nitrate reduction by diatoms and foraminifers is a challenging task that needs particular attention. Knowledge of these genes will not only provide insights into the evolution and biochemistry of dissimilatory nitrate reduction in eukaryotes, but will also enable the development of molecular tools for cultivation-independent investigations directly in the environment. Lessons might be learned from the investigation of denitrifying fungi (Kim et al., 2009; Wei et al., 2015). Several diatom genomes have recently been sequenced, annotated, and interpreted in the context of the cellular nitrogen metabolism (Armbrust et al., 2004; Allen et al., 2006; Bowler et al., 2008). Furthermore, transcriptome sequencing projects of microbial eukaryotes are forthcoming and will provide a rich source of sequence information that can be screened for genes involved in eukaryotic dissimilatory nitrate reduction (Keeling et al., 2014).

The question of dissimilatory nitrate reduction mediated by putative bacterial symbionts in foraminifers (Bernhard et al., 2012a) may also be resolved as soon as eukaryotic and prokaryotic genes for this process can be distinguished. Diatoms and fungi are known to host bacterial endosymbionts too (Foster and Zehr, 2006; Kobayashi and Crouch, 2009), but so far there are no reports on an involvement of these symbionts in dissimilatory nitrate reduction. The relationship between endosymbiotic nitrate reducers and a nitrate-storing eukaryotic host is still enigmatic. It seems paradoxical that a host organism should spend energy to accumulate nitrate intracellularly and then leave it to a bacterial partner without any benefit to itself. In some foraminifers, however, endosymbiotic bacteria are known to use intracellular nitrate for synthesizing amino acids from which the eukaryotic host may benefit (Nomaki et al., 2014, 2015).

The quantitative role of eukaryotic dissimilatory nitrate reduction in the environment is highly uncertain. It has actually been calculated that denitrification by benthic foraminifers equals denitrification by prokaryotes in some marine sediments (Piña-Ochoa et al., 2010a), and calculations are yet to be done for the other eukaryotes. However, the differential measurement of eukaryotic and prokaryotic rates directly in the environment has not yet been achieved due to methodological constraints, and thus novel techniques that capture the uptake and dissimilatory reduction of nitrate in mixed microbial communities *in situ* need to be developed. Additionally, there is no consensus method available for measuring ICNO_3_ pools directly in the environment. It should be evaluated whether freeze-thaw cycling, boiling, whole-core squeezing, and centrifugation are equally efficient in lysing nitrate-storing cells in environmental samples. So far, these techniques also lack selectivity in terms of which taxonomic group contributes how much to the total ICNO_3_ pool.

Given the ubiquitous distribution and high abundance of diatoms, fungi, and foraminifers in marine ecosystems, eukaryotic nitrate storage and dissimilatory nitrate reduction definitely have the potential to contribute significantly to the marine nitrogen cycle. The products of the different pathways of eukaryotic dissimilatory nitrate reduction range from a harmless gas (i.e., dinitrogen) to a strong greenhouse gas (i.e., nitrous oxide) that will escape into the atmosphere. Other products like ammonium or nitrite might be further used by prokaryotes with important roles in nitrogen cycling, such as nitrifiers, denitrifiers, and anammox bacteria. Disentangling the network of nitrogen transformations by eukaryotes and prokaryotes will provide a more comprehensive picture of the marine nitrogen cycle than is currently available.

## Author contributions

This review article was conceived, written, and edited by all authors.

### Conflict of interest statement

The authors declare that the research was conducted in the absence of any commercial or financial relationships that could be construed as a potential conflict of interest.

## Footnotes

^*^note that *G. turgida* was erroneously named *G. pseudospinescens* in Risgaard-Petersen et al. (2006); see Piña-Ochoa et al. (2010a).
