# Supplementary material for: Nitrate Storage and Dissimilatory Nitrate Reduction by Eukaryotic Microbes
Source: Front Microbiol. 2015 Dec 22;6:1492. doi: 10.3389/fmicb.2015.01492 (PMC4686598; doi:10.3389/fmicb.2015.01492)
Supplement: Supplementary file 1 [file Table1.DOCX]

**Table S1: Background information on the source data presented in Figure 4 (“Intracellular nitrate inventories”)**

| **Field site** | **Ecosystem** | **Organisms** | **Source** | **Item** | **Method** |
| --- | --- | --- | --- | --- | --- |
| Gullmar Fjord, Sweden | Fjord sediment | Foraminifers | Risgaard-Petersen et al. 2006 | Fig. 1B | Heat treatment |
| Sagami Bay, Japan | Deep-sea sediment | Foraminifers | Glud et al. 2009 | Fig. 3+5 | Estimated from abundance |
| San Pedro Basin, California | Coastal basin sediment | Foraminifers | Prokopenko et al. 2011 | Fig. 2A | Whole core squeezing |
| Celtic Sea | Marine sand bank | Foraminifers | Larsen et al. 2013 | Fig. 8 | Centrifugation |
| ETSP OMZ, Peru | OMZ sediment | Foraminifers | Glock et al. 2013 | Tab. 4 | Estimated from abundance |
| Hjarbærk Fjord, Denmark | Brackish sediment | Foraminifers Diatoms | Høgslund et al. 2010 | Fig. 6 | Heat treatment^a^ |
| Janssand, German Wadden Sea | Intertidal flat | Foraminifers Diatoms | Marchant et al. 2014 | Text | Freeze-heat cycle^b^ |
| Colne Estuary, U.K. | Estuarine sediment | Foraminifers Diatoms | Papaspyrou et al. 2014 | Fig. 4 | Freeze-thaw cycle^c^  + KCl extraction^d^ |
| Aarhus Bay, Denmark | Coastal bay sediment | Diatoms | Lomstein et al. 1990 | Fig. 2B | Freeze-thaw cycle |
| Cadiz Bay, Spain | Intertidal flat | Diatoms | Garcia-Robledo et al. 2010 | Fig. 1D | Freeze-thaw cycle |
| Dorum, German Wadden Sea | Intertidal flat | Diatoms | Heisterkamp et al. 2012 | Fig. 1 | Freeze-heat cycle |

ETSP = Eastern Tropical South Pacific; OMZ = Oxygen Minimum Zone

^a^Depth range = 0-2 cm, *Thioploca ingrica* was abundant below 2 cm; ^b^Depth range = 0-3 cm; ^c^Average of 3 study sites along the estuary; ^d^KCl extraction mobilizes nitrate adsorbed to sediment particles (i.e., exchangeable nitrate) and hence intracellular nitrate concentrations are overestimated
